# Caveolin-1 regulates hormone resistance through lipid synthesis, creating novel therapeutic opportunities for castration-resistant prostate cancer

**DOI:** 10.18632/oncotarget.10113

**Published:** 2016-06-16

**Authors:** Theodoros Karantanos, Styliani Karanika, Jianxiang Wang, Guang Yang, Masato Dobashi, Sanghee Park, Chengzhen Ren, Likun Li, Spyridon P. Basourakos, Anh Hoang, Eleni Efstathiou, Xuemei Wang, Patricia Troncoso, Mark Titus, Bradley Broom, Jeri Kim, Paul G. Corn, Christopher J. Logothetis, Timothy C. Thompson

**Affiliations:** ^1^ Department of Genitourinary Medical Oncology, The University of Texas MD Anderson Cancer Center, Houston, TX 77030-4009, USA; ^2^ Department of Pathology, The University of Texas MD Anderson Cancer Center, Houston, TX 77030-4009, USA; ^3^ Department of Biostatistics, The University of Texas MD Anderson Cancer Center, Houston, TX 77030-4009, USA; ^4^ Current address: General Internal Medicine Section, Boston Medical Center, Boston University School of Medicine, Boston, MA 02118, USA; ^5^ Current address: Infectious Diseases Division, Warren Alpert Medical School of Brown University, Rhode Island Hospital, Providence, RI 02903, USA

**Keywords:** prostate cancer, caveolin-1, lipid synthesis, mCRPC, FASN

## Abstract

Caveolin-1 (Cav-1) is overexpressed in aggressive and metastatic prostate cancer (PCa) and induces PCa cell proliferation. Androgens mediate lipid synthesis through acetyl-CoA carboxylase-1 (ACC1) and fatty acid synthase (FASN). We investigated the Cav-1-mediated lipid synthesis in the development of castration resistance, and identified novel therapeutic opportunities. Using the PBCre^+^;Pten^loxp/loxp^;PBCav-1^+^ mouse model we found that Cav-1 induction increased cancer incidence and growth, and ACC1-FASN expression in intact and castrated mice. We demonstrated that Cav-1 regulated ACC1 and FASN expression in an AR-independent way and increased palmitate synthesis using western blot analysis, qRT-PCR and mass spectrometry in vitro. By using FASN siRNA and C-75, we found that FASN inhibition was more effective in Cav-1-overexpressing cells. This inhibition was abrogated by ACC1si RNA, revealing the role of malonyl-CoA, an ACC1 product, as a mediator of cytotoxicity. Cav-1 was associated with ACC1 in human tumors and ACC1, FASN, and Cav-1 expression were increased in metastatic PCa compared to primary tumors and normal prostate epithelium. Palmitoleate and oleate levels were higher in BMA from patients with metastatic PCa who responded poorly to abiraterone acetate. Our findings suggest that Cav-1 promotes hormone resistance through the upregulation of ACC1-FASN and lipid synthesis under androgen deprivation, suggesting that FASN inhibition could be used to treat PCa that demonstrates Cav-1 overexpression.

## INTRODUCTION

Metastatic prostate cancer (PCa) remains an incurable disease; it inexorably progresses under androgen deprivation, leading to a lethal state known as metastatic castration-resistant PCa (mCRPC) [1]. The introduction of novel anti-androgens such as abiraterone acetate (AA) and enzalutamide have only slightly increased the survival of chemotherapy-naïve patients with mCRPC [2, 3].

Androgen receptor (AR) remains active during the development of CRPC, indicating that its downstream signaling promotes survival and growth in androgen-depleted conditions. Massie et al demonstrated that AR induces anabolic activities through the upregulation of numerous enzymes, including fatty acid synthase (FASN) and acetyl CoA carboxylase 1 (ACC1), which are implicated in fatty acid synthesis [4, 5]. Moreover, Moon et al found that androgens enhance de novo lipid synthesis in PCa cells [5]. These results, supported by those of other reports [6, 7], suggest that one of the pathways mediating the growth effects of AR is lipid synthesis. Inhibitors of FASN and ACC1, such as C-75 and TOFA, respectively, induce apoptosis and growth arrest in PCa cells [8, 9].

The expression of caveolin-1 (Cav-1), a major component of caveolae implicated in signal transduction and cholesterol transportation, is increased in high-grade and metastatic PCa [10] promoting growth in PCa cells [11, 12]. Nasu et al showed that Cav-1 downregulation increases the sensitivity of mouse PCa cells to androgen depletion [13], while Li et al found that suppression of Cav-1 and castration synergistically inhibited tumor growth in orthotopic models of mouse PCa cells [14]. These results suggest that Cav-1 provides survival and growth advantages upon androgen depletion, promoting the resistance and progression of PCa.

Recent reports have highlighted the role of Cav-1 in anabolic activities, particularly lipid synthesis. Our group found that Cav-1 induces glucose uptake through its interaction with LRP6 and induction of Akt in PCa [15]. According to a report by Di Visio et al, Cav-1 downregulation reduced FASN expression and metastatic potential in a TRAMP mouse model [16], while these 2 molecules interact in PCa cells [17]. Cav-1 and FASN were found to be co-expressed and related to poor prognosis in patients with pancreatic adenocarcinoma [18], and a high-fat diet led to upregulation of Cav-1 and FASN and rapid proliferation of melanoma cells [19]. Finally, Cav-1 expression is positively associated with FASN and ACC1 expression in adipose tissue, further suggesting that it is involved in fatty acid synthesis regulation [20].

The aim of our in vitro and in vivo studies was to evaluate the role of Cav-1 in promoting resistance to hormone therapy by upregulating fatty acid synthesis and maintaining tumor growth and to identify the potential therapeutic opportunities created by targeting fatty acid synthesis in Cav-1 expressing mCRPC.

## RESULTS

### Cav-1 overexpression induced PCa development, enhanced growth, and promoted hormone resistance in PTENcKO tumors

**Figure 1:**
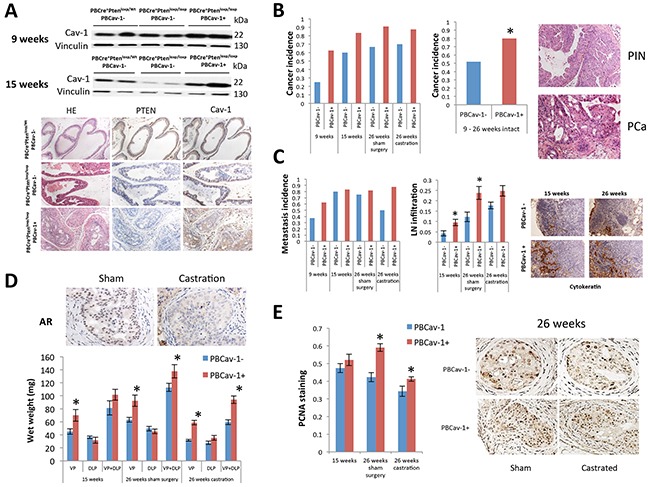
Cav-1 overexpression induced PCa development, enhanced growth, and promoted hormone resistance in PTENcKO tumors **A.** Cav-1 expression in the VPs of 9- and 15–week-old PBCre^+^;Pten^loxp/WT^;PBCav-1^−^, PBCre^+^;Pten^loxp/loxp^;PBCav-1^−^ (PBCav-1^−^), and PBCre^+^;Pten^loxp/loxp^;PBCav-1^+^ (PBCav-1^+^) mice. PBCav-1+ mice had higher expression of Cav-1 in their ventral prostates at 9 (P=0.035) and 15 weeks (P=0.001) of age. Representative PTEN and Cav-1 staining in the VPs of 9-week-old mice. **B.** Incidence of invasive PCa in PBCav-1^−^ (blue columns) and PBCav-1^+^ (red columns) mice. PBCav-1+ mice had a higher incidence of invasive cancer at 9–26 weeks (P=0.036). **C.** Incidence of lymph node metastasis in PBCav-1^−^ (blue columns) and PBCav-1^+^ (red columns) mice. No statistically significant difference was found. However, LN infiltration (percentage of cytokeratin+ metastatic PCa cells) was found to be significantly higher in PBCav-1^+^ than in PBCav-1- mice at 15 (P=0.03) and 26 weeks (P=0.01). **D.** Wet weights of VP, DLP, and VP and DLP combined in PBCav-1^−^ and PBCav-1^+^ mice at 15 weeks and 26 weeks, 10 weeks after sham surgery or castration. PBCav-1^+^ mice had significantly heavier VPs at 15 weeks (P=0.048) and 26 weeks after sham surgery (P=0.002) and after castration (P<0.0001) and heavier VPs and DLPs combined at 26 weeks after sham surgery (P=0.043) and castration (P=0.00034). Representative images from AR staining after sham surgery and after castration show that AR nuclear localization was significantly reduced 10 weeks after castration compared to sham surgery **E.** PCNA staining of cells from PBCav-1^−^ and PBCav-1^+^ mice at 15 weeks and 26 weeks after sham surgery or castration. PBCav-1^+^ mice had a higher percentage of PCNA+ cells in their VPs at 26 weeks after sham surgery (P=0.0013) and castration (P=0.025).

It has been shown by our group that Cav-1 promotes the survival and growth of PCa cells while Cav-1 knockdown enhances the effects of androgen depletion to suppress orthotopic PCa xenograft growth and metastatic potential in vivo [14]. These results supported that Cav-1 induces the PCa growth and suggested a possible implication of Cav-1 in the development of resistance to androgen depletion and CRPC. To study the effects of Cav-1 overexpression on castration-induced growth suppression in vivo, we generated a transgenic animal model by crossing PBCre^+^;Pten^loxp/loxp^ mice with PBCav-1^+^ mice (Supplementary Figure S1); the latter model has increased incidence of epithelial hyperplasia through inducible Cav-1 expression in the ventral (VP) and dorsolateral prostate (DLP) [21]. Initially, we collected the VPs and DLPs of PBCre^+^;Pten^loxp/WT^;PBCav-1^−^, PBCre^+^;Pten^loxp/loxp^;PBCav-1^−^, and PBCre^+^;Pten^loxp/loxp^;PBCav-1^+^ mice at 9 and 15 weeks, performed sham surgery or castration at 16 weeks, and collected the VPs and DLPs at 26 weeks of age. We examined Cav-1 expression in the VPs and DLPs of these mice at 9 and 15 weeks using Western blot analysis. PBCre^+^;Pten^loxp/loxp^;PBCav-1^+^ (PBCav-1^+^) mice expressed Cav-1 at significantly higher levels in the VPs (P=0.035-9 weeks, P=0.001-15 weeks) than did PBCre^+^;Pten^loxp/loxp^;PBCav-1^−^ (PBCav-1^−^) mice, on the basis of a Western blot analysis (Figure 1A). The difference in Cav-1 expression was not statistically significant in the DLPs (P=0.2). Thus, we focused mainly on the data derived from VPs.

**Figure 2:**
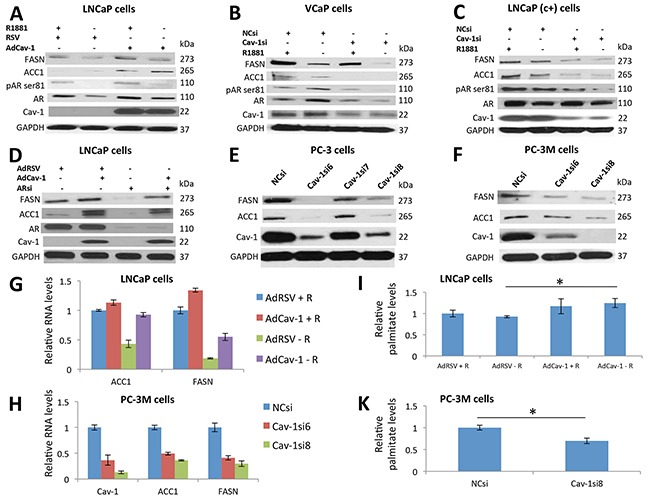
Cav-1 regulated ACC1 and FASN expression in PCa cells in an AR-independent manner at the transcriptional level, promoting palmitate synthesis **A.** Androgen deprivation decreased the phosphorylation of AR, AR, and FASN protein levels in LNCaP cells. Cav-1 induction increased the phosphorylation of AR, AR, ACC1, and FASN protein levels in the presence and absence of R1881. **B.** Androgen deprivation increased the phosphorylation and total protein levels of AR and decreased the FASN and ACC1 protein levels in VCaP cells. Downregulation of Cav-1 decreased the phosphorylation and total levels of AR in the presence and absence of androgens. In addition, downregulation of Cav-1 decreased the protein levels of FASN and ACC1 synergistically with androgen deprivation. **C.** Androgen deprivation decreased the phosphorylation and protein levels of AR but only mildly affected the FASN and ACC1 protein levels in LNCaP (c+) cells. Cav-1 knockdown decreased the phosphorylation and protein levels of AR synergistically with androgen deprivation and significantly decreased the FASN and ACC1 protein levels. **D.** Induction of Cav-1 increased the protein levels of FASN and ACC1, and knockdown of AR decreased the levels of these proteins in LNCaP cells. Induction of Cav-1 in the absence of AR increased the levels of these proteins, suggesting that Cav-1 promotes the expression of FASN and ACC1 independently of AR. **E.** Cav-1 knockdown decreased expression of ACC1 and FASN in PC-3 cells. **F.** Downregulation of Cav-1 led to decreased protein levels of ACC1 and FASN in PC-3M cells. **G.** In the presence of androgens Cav-1 induction increased the RNA levels of FASN (p<0.001) in LNCaP cells. In the absence of androgens, Cav-1 induction increased the RNA levels of ACC1 (p<0.001) and FASN (p=0.026). **H.** The downregulation of Cav-1 led to significantly lower RNA levels of ACC1 (P<0.001 for both Cav-1siRNA6 and Cav-1siRNA8) and FASN (P<0.001 for both Cav-1siRNA6 and Cav-1siRNA8) in PC-3M cells. **I.** Palmitate measurements showed that in the absence of androgens, Cav-1 induction increased the levels of palmitate (P=0.041) in LNCaP cells. **J.** Cav-1 knockdown significantly reduced palmitate levels (P=0.01) in PC-3M cells.

Next, we examined the incidence of invasive PCa in the VPs and found that PBCav-1^+^ mice had a higher incidence of invasive disease at 9, 15, and 26 weeks after sham surgery or castration than PBCav-1^−^ mice did (Figure 1B). Invasive cancer was defined as PCa spread beyond the basal membrane of the prostate epithelium. Focusing only on intact mice, we found that the invasive PCa incidence was 1.5 times higher in PBCav-1^+^ compared to PBCav-1^−^ mice from 9 to 26 weeks of age (P=0.036) (Figure 1B). Moreover, the PBCav-1^+^ mice demonstrated a trend for higher incidence of lymph node metastasis without reaching statistical significance (16/25 PBCav-1- mice vs 19/25 PBCav-1^+^ mice P=0.26) (Figure 2C). To evaluate the extension of metastatic disease, we measured the percentage of metastatic cancer cells in lymph nodes based on cytokeratin staining. We found that intact PBCav-1^+^ mice had a significantly higher percentage of metastatic cells in their lymph nodes at 15 (P=0.03) and 26 weeks (P=0.01) than PBCav-1^−^ mice did (Figure 2C). These data suggest that Cav-1 overexpression promoted PCa progression in a *PTEN*-deleted transgenic model. Next, we examined the wet weight of VP, DLP, and the VP and DLP combined (VP + DLP) at 15 weeks and 26 weeks after sham surgery or castration. We found that PBCav-1^+^ mice had significantly heavier VPs at all these time points (P=0.048-15 weeks, P=0.002-26 weeks, P<0.0001-26 weeks) and heavier VP + DLP at 26 weeks after sham surgery (p=0.043) and castration (P=0.00034) compared to PBCav-1^−^ mice. Particularly, PBCav-1^+^ mice have 1.6 heavier VP+DLP compared to PBCav-1^−^ at 26 weeks after castration (Figure 1D). To further support our findings, we evaluated the proliferation index of PCa cells in these tumors using proliferation cell nuclear antigen staining (PCNA). We found that PBCav-1^+^ mice had a significantly higher percentage of PCNA+ PCa cells in their VPs at 26 weeks after sham surgery (P=0.0013) and castration (P=0.025) than PBCav-1^−^ mice did (Figure 1E). These data suggest that Cav-1 expression promotes PCa growth and progression in the presence and absence of androgens in a *PTEN*-deleted model.

### Cav-1 regulated ACC1 and FASN expression in PCa cells in an AR-independent manner at the transcriptional level, promoting palmitate synthesis

We then evaluated the hypothesis that Cav-1 regulates the expression of ACC1 and FASN, particularly under androgen depletion. Cav-1 induction increased ACC1 and FASN levels in the presence (P=0.01-ACC1, P=0.023-FASN) and, absence of androgens (P<0.001-ACC1, P=0.016-FASN) (Supplementary Figure S2). Moreover, Cav-1 induction increased phosphorylation and AR levels in the presence (P=0.02-pAR ser81, P=0.021-AR) and, absence of androgens (P=0.01-pAR ser81, P=0.012-AR) (Supplementary Figure 2, 3). A synergistic effect of AdCav-1 combined with androgens was found for AR phosphorylation levels (P=0.0012) (Supplementary Figure S2) (Figure 2A).

We then knocked-down Cav-1 expression in VCaP and LNCaP (c+) cells in the presence and absence of androgens. Androgen depletion increased AR levels in VCaP cells (P<0.001) (Supplementary Figure S2) (Figure 2B), which was consistent with the results of a previous study [22]. Androgen depletion increased AR phosphorylation (P=0.002) (Supplementary Figure S2) (Figure 2B). These effects were at least partially mediated by Cav-1 since Cav-1 downregulation abolished them (P<0.001-AR, P=0.08-pAR ser81) (Supplementary Figure S2) (Figure 2B). As expected, androgen depletion and Cav-1 knockdown reduced the levels of ACC1 and FASN (P=0.03, P<0.001, respectively, for androgen depletion and P<0.001, P=0.003, respectively, for Cav-1 knockdown), whereas the combination of these 2 treatments had synergistic effects on ACC1 reduction (P=0.049) and additive effects on FASN reduction (Supplementary Figure S2) (Figure 2B). Similarly, based on our Western blot data, Cav-1 downregulation reduced ACC1 (P<0.001) and FASN (P=0.002) levels in LNCaP (c+) cells, especially under androgen depletion (P<0.001-ACC1 and FASN) (Supplementary Figure S2) (Figure 2C). Of note, we found that Cav-1si and androgen deprivation had synergistic effects on ACC1 level reduction (P=0.0085) (Supplementary Figure S2).

AR downregulation reduced ACC1 and FASN protein levels (P<0.001-ACC1 and FASN), but Cav-1 induction increased them (P<0.001-ACC1, P=0.025-FASN), even after AR downregulation (P<0.001-ACC1 and FASN) (Supplementary Figure S2) (Figure 2D). To Further support that the effect of Cav-1 on ACC1 and FASN is AR- independent, we downregulated Cav-1 in AR- PC-3 and PC-3M cells. Cav-1 downregulation led to reduced ACC1 (P<0.01) and FASN expression (P<0.001-PC-3 cells, P=0.002 and P<0.001-PC-3M cells) in both cell lines (Supplementary Figure S2) (Figure 2E, 2F).

We then induced Cav-1 expression in LNCaP cells in the presence and absence of androgens and performed qRT-PCR; in the absence of androgens, the RNA levels of ACC1 were 2.2 times higher in AdCav-1 transfected cells compared to AdRSV transfected cells (P<0.001) (Figure 2G). We also demonstrated that in the presence and absence of androgens, the RNA levels of FASN were 1.3 and 2.9 times higher respectively in cells with Cav-1 upregulation (P<0.001-presence of androgens, P=0.0026- absence of androgens) (Figure 2G). In PC-3M cells, Cav-1 downregulation led to significantly lower RNA levels of ACC1 (P<0.001-both Cav-1siRNA6 and Cav-1siRNA8, 2 and 2.7 times lower respectively) and FASN (P<0.001-both Cav-1siRNA6 and Cav-1siRNA8, 2.4 and 3.3 times lower, respectively) (Figure 2H). We then measured the levels of palmitate, the final product of the lipid synthesis pathway, after manipulating Cav-1 expression and androgen levels. Cav-1 induction significantly increased palmitate levels in the absence of androgens (P=0.041) (Figure 2I). Downregulation of Cav-1 in PC-3M cells led to decreased palmitate levels (P=0.01) (Figure 2J). Overall, these results suggest that Cav-1 induced ACC1 and FASN expression, activating fatty acid synthesis in PCa cells under androgen-depleted conditions, through an AR-independent mechanism, acting mainly at the transcriptional level.

### PBCav-1^+^ mice expressed higher ACC1 and FASN levels and castration increased Cav-1 and ACC1 expression in PTENcKO tumors

**Figure 3:**
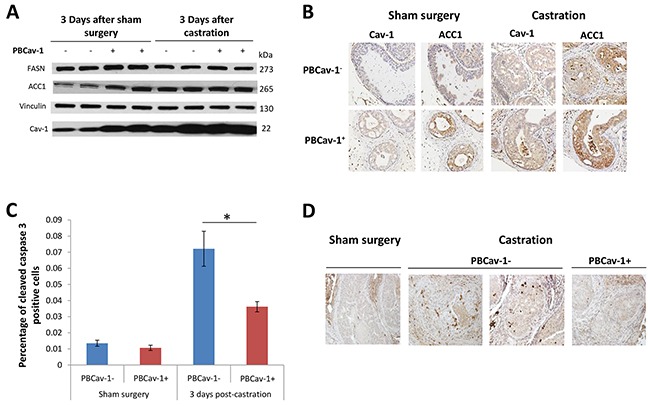
PBCav-1+ mice expressed higher ACC1 and FASN levels, and castration increased Cav-1 and ACC1 expression in PTENcKO tumors **A.** 3 days after sham surgery, PBCav-1^+^ mice had higher expression of FASN (P=0.011) and ACC1 (P=0.002) than did PBCav-1^−^ mice. Mice that had undergone castration had higher expression of Cav-1 than did mice that had undergone sham surgery (P=0.014 for PBCav-1^−^ mice and P=0.035 for PBCav-1^+^ mice). In addition, after castration, PBCav-1^−^ mice had higher protein expression of ACC1 than did PBCav-1^−^ mice after sham surgery (P=0.011). **B.** Cav-1 and ACC1 staining intensity was found to be significantly higher in animals who had undergone castration than in those who had undergone sham surgery (P=0.017 for Cav-1 and P=0.0077 for ACC1). The staining intensities of Cav-1 and ACC1 were found to be correlated in the examined tumors by Spearman correlation (P<0.025). **C.** PBCav-1^+^ mice had a significantly lower percentage of cleaved caspase 3+ cancer cells (P=0.025) 3 days after castration. **D.** Representative slides from the VPs of PBCav-1^−^ and PBCav-1^+^ mice 3 days after sham surgery or castration showing cleaved caspase 3 staining.

Next, we examined the early effects of castration on *PTEN*-deleted tumors, focusing on the expression of ACC1 and FASN. We performed sham surgery or castration in PBCav-1^−^ and PBCav-1^+^ mice at 16 weeks and collected the VPs 3 days after surgery. We found that intact PBCav-1^+^ mice expressed higher levels of ACC1 and FASN than PBCav-1^−^ mice did (P=0.002, P=0.011, respectively) (Figure 3A). Cav-1 expression in the VPs of PBCav-1^−^ and PBCav-1^+^ mice was higher 3 days after castration than after sham surgery, according to Western blot data (P=0.014-PBCav-1^−^ mice, P=0.035-PBCav-1^+^ mice) (Figure 3A). Of note, castration increased ACC1 protein levels in PBCav-1^−^ mice (P=0.016) (Figure 3A).

Next, we examined the staining intensity of Cav-1 and ACC1 in intact and castrated PBCav-1^−^ and PBCav-1^+^ mice. Castrated animals had higher levels of Cav-1 and ACC1 (P=0.017, P=0.0077, respectively), whereas Cav-1 and ACC1 were found to be correlated in the examined tissues (r=0.54, P<0.025) (Figure 3B). Finally, to assess the survival benefit of Cav-1 expression in PTENcKO tissues, we evaluated the apoptotic effects of castration compared to sham surgery. At 3 days after castration, the percentage of cleaved caspase-3+ cancer cells in the VPs of PBCav-1^−^ mice was significantly higher than in those of PBCav-1^+^ mice (P=0.025) (Figure 3C). Overall, these data suggest that Cav-1 induction was related to ACC1 and FASN expression in PTENcKO tissues and decreased the apoptotic effects of castration, which upregulated Cav-1 and ACC1, suggesting that this pathway is critical for the survival and proliferation of PCa cells under androgen depletion.

### FASN was critical for the survival of PCa cells expressing Cav-1 under androgen deprivation through tumor growth and resistance effects

**Figure 4:**
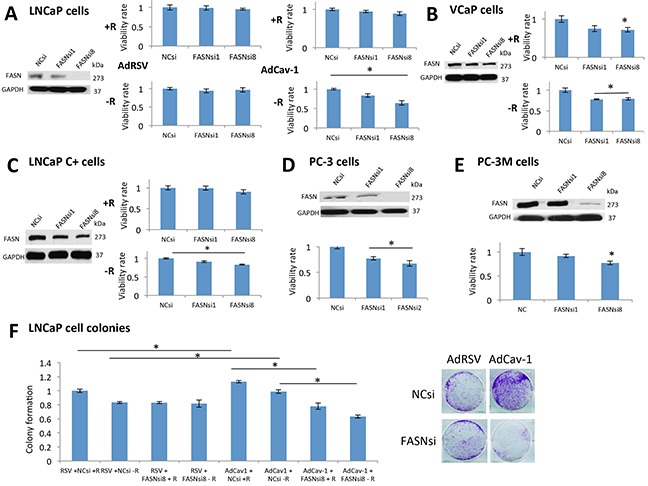
FASN was critical for the survival of PCa cells expressing Cav-1 under androgen deprivation, mediating its growth and resistance effects **A.** According to the results of an MTS assay, neither of the siRNAs affected the survival of LNCaP cells treated with AdRSV in the presence or absence of androgens. On the contrary, when cells were treated with AdCav-1, both siRNAs reduced the survival of LNCaP cells in the absence of androgens (P=0.031 for FASNsiRNA1 and P=0.03 for FASNsiRNA8). **B.** In the presence of R1881, only FASNsiRNA8 reduced the survival of VCaP cells (P=0.036), whereas in the absence of R1881, both siRNAs reduced the survival of these cells (P=0.007 for FASNsiRNA1 and P=0.014 for FASNsiRNA8). **C.** In the presence of R1881, the siRNAs had no effect on the survival of LNCaP (c+) cells, whereas in the absence of R1881, both siRNAs reduced the survival of these cells (P=0.035 for FASNsiRNA1 and P=0.0005 for FASNsiRNA8). **D.** Both siRNAs significantly reduced the survival of PC-3 cells (P=0.013 with FASNsiRNA1 and P=0.009 with FASNsiRNA8). **E.** FASNsiRNA8 significantly reduced the survival of PC-3M cells (P=0.035). As seen in a Western blot analysis, FASNsiRNA1 was not effective at downregulating FASN in PC-3M cells. **F.** Cav-1 induction increased the growth of LNCaP cells in the presence (P=0.026) and absence (P=0.002) of R1881. LNCaP cells treated with AdRSV were not significantly affected by FASN downregulation in the presence and absence of R1881. After treatment with AdCav-1, FASN downregulation significantly reduced the number of colonies in the presence (P=0.0027) and absence of R1881 (P=0.002).

We then tested the hypothesis that inhibition of the ACC1-FASN pathway should abrogate the growth effects of Cav-1. Importantly, FASN inhibition can be toxic through the accumulation of malonyl-CoA [23], produced by ACC1 which is upregulated in Cav-1 expressing cells. We induced Cav-1 and downregulated FASN in LNCaP cells in the presence and absence of androgens. Cells without Cav-1 induction did not demonstrate reduced survival under FASN downregulation, in the presence or absence of androgens (Figure 4A). On the contrary, after Cav-1 induction, FASN downregulation significantly reduced cells' survival after androgen depletion (P=0.031-FASNsiRNA1 and P=0.003-FASNsiRNA8) (Figure 4A). FASN downregulation significantly reduced VCaP cells' survival in the presence of androgens with FASNsiRNA8 (P=0.036) and in their absence (P=0.007-FASNsiRNA1 and P=0.014-FASNsiRNA8) (Figure 4B). In LNCaP (c+) cells, FASN downregulation reduced their survival only in the absence of androgens (P=0.035-FASNsiRNA1 and P=0.0005- FASNsiRNA8) (Figure 4C). Regarding the PC-3 and PC-3M cells, inhibition of FASN significantly reduced the survival of PC-3 (P=0.013- FASNsiRNA1, P=0.009-FASNsiRNA8) (Figure 4D) and PC-3M cells (P=0.035- FASNsiRNA8) (Figure 4E).

We then performed colony assays in LNCaP cells, inducing Cav-1 in the presence or absence of androgens, with or without FASN downregulation. LNCaP cells without Cav-1 induction were not significantly affected by FASN downregulation in the absence of androgens (Figure 4F). On the contrary, Cav-1 induction increased the colony formation in the absence of androgens (P=0.002), but the downregulation of FASN significantly reduced the number of colonies in the presence (P=0.0027-FASNsiRNA8) and absence (P=0.002-FASNsiRNA8) of androgens (Figure 4F). Overall, these results suggest that Cav-1 expression rendered PCa cells sensitive to FASN inhibition.

### Cav-1 upregulation increases the sensitivity of PCa cells to the FASN inhibitor C-75

**Figure 5:**
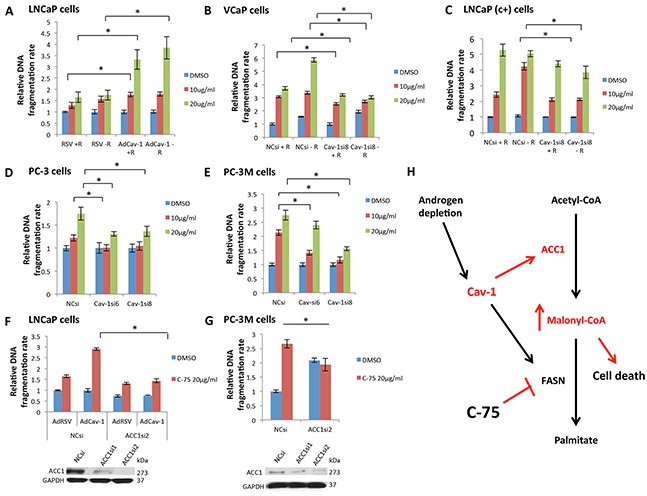
Cav-1 expression increased the sensitivity of PCa cells to the FASN inhibitor C-75, leading to cell death through malonyl-CoA accumulation **A.** Cav-1 induction increased the apoptotic effect of C-75 in LNCaP cells in the presence (P=0.021 for 10 μg/ml and P=0.004 for 20 μg/ml) and absence of R1881 (P=0.008 for 20 μg/ml). **B.** Cav-1 downregulation reduced the apoptotic effect of C-75 in VCaP cells in the presence (P=0.0066 for 10 μg/ml and P=0.0054 for 20 μg/ml) and absence of R1881 (P<0.0001 for 10 μg/ml and P=0.026 for 20 μg/ml). **C.** Cav-1 downregulation reduced the apoptotic effect of C-75 in LNCaP (c+) only in the absence of R1881 (P=0.00014 for 10 μg/ml and P=0.036 for 20 μg/ml). **D.** Cav-1 downregulation reduced the apoptotic effect of C-75 at 10 μg/ml using Cav-1siRNA6 (P=0.018) and at 20 μg/ml using Cav-1siRNA6 and Cav-1siRNA8 (P=0.007 and P=0.02, respectively) in PC-3 cells. **E.** Cav-1 downregulation reduced the apoptotic effect of C-75 at 10 μg/ml (P=0.001 for Cav-1siRNA6 and P<0.001 for Cav-1siRNA8) and at 20 μg/ml (P<0.001 for Cav-1siRNA8) in PC-3M cells. **F.** LNCaP cells that had been pretreated with ACC1siRNA2 were less sensitive to C-75 (P<0.001) than were cells treated with NCsi. **G.** PC-3M cells that had been pretreated with ACC1siRNA2 were less sensitive to C-75 (P=0.014) than were cells treated with NCsi. **H.** Cav-1, which is induced in androgen-depleted conditions, promoted ACC1 and FASN expression. FASN inhibition by C-75 in the setting of Cav-1 upregulation led to the accumulation of the intermediate product of ACC1, malonyl-CoA, which is cell toxic proportionally to its accumulation.

We used the chemical inhibitor of FASN, C-75 [24], to further demonstrate that inhibition of FASN can be highly toxic upon Cav-1 induction. We found that in the presence of androgens, LNCaP cells overexpressing Cav-1 were more 1.4 times more sensitive to 10 (P=0.021) and 2 times more sensitive to 20 μg/ml (P=0.004) C-75. In the absence of androgens, Cav-1-overexpressing LNCaP cells were 2.25 times more sensitive to 20 μg/ml C-75 (P=0.008) (Figure 5A).

VCaP cells treated with Cav-1siRNA8 were less sensitive to 10 and 20 μg/ml C-75 in the presence (P=0.0066, P=0.0054) and absence of androgens (P=0.026, P<0.0001) (Figure 5B). Particularly, Cav-1siRNA treatment decreased the sensitivity of VCaP cells to 20 μg/ml C-75 by almost 2 times in the absence of androgens. Cav-1 knockdown reduced LNCaP (c+) cells' sensitivity to 10 and 20 μg/ml C-75, only in the absence of androgens (P=0.00014, P=0.036) (Figure 5C). Of note, cells treated with the Cav-1siRNA8 were 2 times less sensitive to 10 μg/ml C-75 in the absence of androgens. Cav-1 knockdown in PC-3 cells significantly reduced the cells' sensitivity to 10 (P=0.018 with Cav-1siRNA6) and 20 μg/ml C-75 (P=0.007 with Cav-1siRNA6, P=0.02 with Cav-1siRNA8) (Figure 5D). Finally, PC-3M cells treated with Cav-1siRNA6 and Cav-1siRNA8 were less sensitive to 10 μg/ml (P=0.001, P<0.001 respectively) and 20 μg/ml C-75 (P<0.001 with Cav-1siRNA8) (Figure 5E). Particularly, Cav-1siRNA8 treatment decreased the sensitivity to 10 and 20 μg/ml C-75 by almost 2 times.

To confirm that the increased sensitivity of Cav-1-overexpressing PCa cells to C-75 was associated with ACC1 induction and accumulation of its product, malonyl-CoA, we induced Cav-1 and downregulated ACC1 in LNCaP cells. Cav-1 induction increased the apoptotic effect of C-75 (P<0.001) (Figure 5F). Concurrent ACC1 downregulation reduced the apoptotic effect of C-75 (P<0.001) (Figure 5F), abrogating the effects of AdCav-1. Similarly, ACC1 knockdown in PC-3M cells significantly reduced the apoptotic effect of C-75 (P=0.014) (Figure 5G). According to these results, the increased sensitivity of cells to C-75 with Cav-1 overexpression is mediated by ACC1 upregulation.

On the basis of our findings, Cav-1 was induced in androgen-depleted conditions, and promoted ACC1 and FASN protein expression; this induced palmitate production, which mediates anabolic synthesis and cell proliferation. FASN inhibition by C-75 was particularly toxic in cells that overexpressed Cav-1, probably due to the accumulation of the intermediate cytotoxic product, malonyl-CoA produced by ACC1 (Figure 5H).

### Cav-1/ACC1/FASN signaling is induced in primary and metastatic PCa and elevated levels of palmitoleate and oleate in the bone marrow aspirates from mCRPC were associated with a poor response to AA

**Figure 6:**
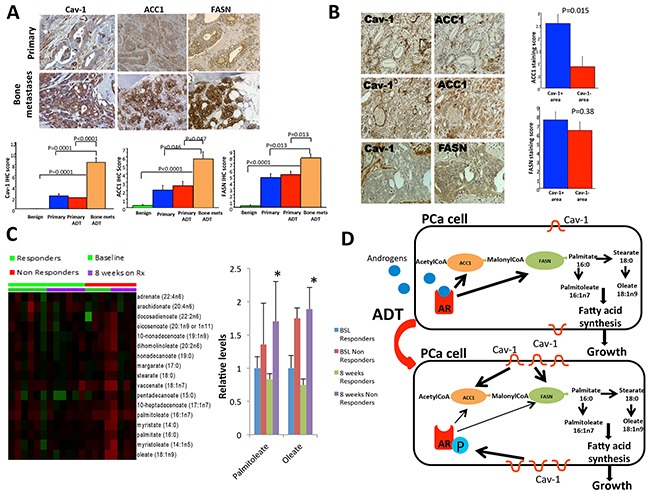
Cav-1, ACC1 and FASN expression in human primary prostate tumors and bone metastases Cav-1 expression was associated with ACC1 expression and elevated levels of palmitoleate and oleate in the bone marrow aspirates from mCRPC were associated with worse response to abiraterone acetate. **A.** Cav-1, ACC1and FASN expressions are significantly increased in metastatic PCa compared to primary treated and untreated PCa. P-values are shown in the figure. **B.** Left panel: Cav-1- PCa cells had minimal level of ACC1 (top row); the Cav-1+ cancer cells exhibited strong ACC1 immunostaining (middle row), whereas FASN immunostaining was not reduced in the area with low Cav-1 (bottom row). Original magnification: 200x. Right panel: the average ACC1 immunostaining score in the Cav-1+ PCa was significantly higher than that in the Cav-1–area of the same PCa tissue (P=0.015); data are plotted as means± SEM. P value was from by paired sign test. **C.** Long-chain fatty acids were measured in bone marrow aspirations from patients with mCRPC treated with Abiraterone Acetate (AA). Two products of palmitate (palmitoleate and oleate) were found to be higher at 8 weeks of treatment in non-responders to AA compared to responders (p=0.015-palmitoleate and p=0.045-oleate). **D.** Schematic summary of our findings: AR is activated by androgens and promotes the expression of ACC1 and FASN, which mediate the synthesis of long-chain fatty acids including palmitoleate and oleate leading to PCa cell growth. Under androgen deprivation therapy (ADT), Cav-1 is upregulated and phosphorylates AR on the serine 81 residue. Cav-1 upregulation also increases the expression of ACC1 and FASN, promoting fatty acid synthesis and PCa cell growth under conditions of androgen deprivation.

To further support the implication of Cav-1/ACC1/FASN signaling in the progression of PCa we performed Cav-1, ACC1 and FASN immunostaining in normal prostate tissues, primary untreated and ADT treated PCa and metastatic disease. We demonstrated that there is increasing Cav-1, ACC1 and FASN expression during prostate cancer progression (P<0.0001, P=0.007 or P<0.0001 respectively based on Fisher's exact test). Cav-1, ACC1and FASN expression is significantly increased in metastatic PCa compared to primary treated and untreated PCa (P=0.0001 and P<0.0001 for Cav-1, P=0.046 and P=0.047 for ACC1 respectively, P=0.013 and p=0.013 for FASN respectively) (Figure 6A).

We then focused on identifying correlations between Cav-1 and ACC1/FASN in our samples. Focusing on primary tumors, 7 of the 30 specimens were Cav-1+, 22 were ACC1+ and 29 were FASN+. There was no significant correlation between the three proteins in both primary tumors and bone metastases (all P >0.05, Spearman correlation test). However, since Cav-1 expression was usually focal in human primary PCa [25] and ACC1 immunostaining levels varied among cancer cells, we compared the ACC1 levels in the Cav-1+ and Cav-1- areas in the Cav-1+ PCa specimens (Figure 6B). The ACC1 immunostaining score in the Cav-1+ areas was significantly higher than that in Cav-1- areas (p=0.0146), further supporting the association of Cav-1 expression with ACC1 in PCa (Figure 6B). FASN was broadly expressed in the cancer cells. The difference in FASN levels between the Cav-1+ and Cav-1- areas in the Cav-1+ PCa specimens was not statistically significant (p=0.38) (Figure 6B). Regarding the metastatic tissues, the correlation between Cav-1 and ACC1/FASN was not found to be statistically significant but it should be noted that only 8 patients were available for this analysis.

We then determined whether the ACC1/FASN pathway and its downstream products were related to response to androgen depletion in a human model of PCa. We included bone marrow aspirates (BMAs) from patients with mCRPC who were treated with AA which inhibits androgen biosynthesis in the tumor microenvironment [2] and is expected to create a condition of androgen depletion in metastatic sites. We analyzed the levels of long-chain fatty acids, at baseline and at 8 weeks on AA. We included BMAs from 6 responders to AA and 4 non-responders. Responders were considered those patients who remained longer than 7.6 months under treatment since this was the median time on treatment among patients in the study.

Dehydroisoandrosterone sulfate (DHEA-S) levels in BMAs were significantly lower at 8 weeks compared to the baseline measurements (p<0.001- responders, p=0.029- non-responders) without significant differences between responders and non-responders. A BMA analysis demonstrated that the levels of 2 long-chain fatty acids, palmitoleate and oleate, were significantly higher in non-responders' BMAs than in responders' at 8 weeks (p=0.015-palmitoleate, p=0.047-oleate) (Figure 6C). These 2 metabolites are downstream products of palmitate in the fatty acid synthesis pathway (Figure 6D). These results suggest that fatty acid synthesis is a critical pathway during the development of resistance to androgen depletion.

## DISCUSSION

We report here the generation of a transgenic animal model that combined prostate-specific *PTEN* deletion and Cav-1 overexpression, and showed that Cav-1 upregulation led to a higher incidence of invasive PCa, and increased VP wet weight and cellular proliferation under androgen-depleted conditions.

We showed that Cav-1 induction promoted ACC1 and FASN expression in AR+ PCa cells and increased AR phosphorylation and protein levels. Serine 81 phosphorylation of AR is strongly associated with PCa growth and proliferation and induces AR transcriptional activity [22, 26]. Cav-1 increases AR nuclear translocation [11], while its overexpression induces AR phosphorylation on serine 210, which is associated with increased activation under androgen-stimulated conditions [27]. We found that Cav-1 induced the expression of ACC1 and FASN, even in the absence of AR, and in AR- PCa cell lines and that Cav-1 manipulation resulted in alteration of ACC1 and FASN RNA levels and palmitate synthesis. These data demonstrate that Cav-1 regulated ACC1-FASN signaling independently of AR at the transcriptional level, activating fatty acid synthesis in PCa cells.

We extended our findings in the PTENcKO model showing that Cav-1 induction was associated with higher levels of ACC1 and FASN; castration led to upregulation of Cav-1 and ACC1, suggesting that this pathway provided a survival advantage for the PCa cells. Our group previously found that Cav-1 induction promotes hormone resistance in mouse PCa cells [13] and that Cav-1 inhibition and castration synergistically inhibit tumor growth in orthotopic models of these cells [28], but to our knowledge, this is the first report showing that Cav-1 upregulation promoted tumor growth under androgen deprivation in a transgenic mouse model and that castration increased its expression. More important is the novel finding that Cav-1 and ACC1 levels were associated with one another in a cancerous transgenic model of PCa, further supporting the important role of Cav-1 as a regulator of the fatty acid synthesis pathway.

C-75, a FASN inhibitor, has been studied as a novel therapeutic agent in PCa cells [29]; recent reports have shown that FASN inhibition leads to malonyl-CoA accumulation, with toxic effects in cancer cells [23, 24, 29]. Given that FASN activity is the last step in the lipid synthesis pathway and Cav-1 induces both ACC1 and FASN, we hypothesized that the FASN inhibition in Cav-1 overexpressing cells would lead to the accumulation of malonyl-CoA, resulting in an intense apoptotic effect. Indeed, we found that FASN downregulation reduced the survival and growth advantage of PCa cells expressing Cav-1, while Cav-1- cells were not sensitive to this genetic manipulation. This finding suggests that the resistance pathway initiated by Cav-1 expression can be inhibited by targeting FASN, and that cells overexpressing Cav-1 are particularly sensitive to this approach. Finally, Cav-1 expression was strongly associated with increased sensitivity to C-75, which is mediated by ACC1, since its downregulation abrogated Cav-1's effect on C-75 toxicity. These novel findings encompass an interesting therapy consideration for mCRPC; the development of resistance to hormone therapy leads to loss of AR signaling as a viable therapy target, but at the same time, Cav-1, which may supplant specific AR functions in mCRPC, can regulate growth through the Cav-1-ACC1-FASN pathway.

Moving from the in vitro and in vivo setting to clinical samples in an effort to confirm our concept in patients, we found that Cav-1, ACC1 and FASN expression is significantly increased in metastatic disease compared to primary PCa and normal prostate further supporting that this signaling is upregulated during PCa progression. ACC1 expression was significantly and proportionally higher in Cav-1+ areas in primary prostate tumors than in Cav-1-areas; this is a novel finding since the only known association between Cav-1 and ACC1 has been documented in adipose tissue [20]. Moreover, this association is particularly important for the introduction of FASN inhibition as a therapeutic approach for Cav-1-expressing tumors since its efficiency is mediated by ACC1 induction. We also measured long-chain fatty acids in the BMAs of patients with mCRPC under AA showing that the downstream products of palmitate synthesis, palmitate oleate and palmitoleate, were increased in non-responders to AA at 8 weeks after the initiation of treatment. This finding suggests that de novo fatty acid synthesis is implicated in tumor growth under androgen-independent conditions. On the basis of these results, we suggest that Cav-1-ACC1-FASN represents critical signaling for the survival and growth of PCa under androgen deprivation.

Patients with metastatic PCa will eventually develop resistance to AR inhibition and their survival at this stage of disease is poor. Our results support that Cav-1 promotes tumor growth under hormone therapy through the upregulation of ACC1-FASN. FASN inhibition can target this pathway efficiently, providing new treatment options for mCRPC. Thus, although Cav-1 is associated with the development of resistance to hormone therapy, its upregulation can be exploited therapeutically via concurrent targeting of its downstream pathway. Overall, consideration of the results of our study may contribute to the development of novel therapy approaches in which the mechanism of resistance to one treatment may render cancer cells particularly sensitive to other treatments.

## MATERIALS AND METHODS

### Cell lines and reagents

The human PCa cell lines LNCaP and PC-3 were obtained from American Type Culture Collection and PC-3M was a generous gift from Dr. Isaiah Fidler (MD Anderson Cancer Center). The cell lines were grown and authenticated as previously reported [30]. LNCaP (c+), a Cav-1-expressing LNCaP variant, was obtained during the propagation of LNCaP by our group [11]. Adenovirus-mediated gene transduction was performed as previously described [12]. C-75 (10005270) was purchased from Cayman.

### RNA interference

Cav-1siRNA6 (SI00299614), Cav-1siRNA7 (SI00 299621), Cav-1siRNA8 (SI00299628), FASNsiRNA1 (SI00059752), FASNsiRNA8 (SI00059759), ACACA siRNA1 (SI00013622), ACACAsiRNA2 (SI00013629), and NCsiRNA (1022076) were purchased from Qiagen; ARsiRNA (s1538) was purchased from Life Technologies. Description of the Cav-1, FASN, ACC1 and AR knockdown is presented in the Supplementary Materials and Methods.

### Antibodies for western blotting analysis

Antibodies against Cav-1 (sc-894), AR (sc-816), and GAPDH (sc-25778) were purchased from Santa Cruz. Antibodies against FASN (#3180) and ACC1 (#3662) were purchased from Cell Signaling. The antibody against pAR ser81 (04-078) was purchased from Millipore, and the antibody against vinculin (V9131) was purchased from Sigma-Aldrich.

### qRT-PCR primers

TaqMan primer was purchased from Qiagen for GAPDH (catalog number: 402869), and Cyrb green primers were purchased from Qiagen for Cav-1 (catalog number: PPH00739A-200), ACC1 (catalog number: PPH02316A-200), and FASN (catalog number: PPH01012B-200).

### Cell viability and DNA fragmentation assays

Cell viability was analyzed using an MTS CellTiter 96 AQueous One Solution cell proliferation assay (Promega, Madison, WI) as previously described [31]. A DNA fragmentation analysis was performed using a Cell Death Detection ELISA (Roche Applied Science, Indianapolis, IN) as previously described [32].

### Colony formation assay

PCa cells were treated with Cav-1si, ACC1si, FASNsi, or NCsi and AdRSV or AdCav-1, respectively. Cells were trypsinized 24 hours later, reseeded into 6-well plates at low density (VCaP: 1.0 × 10^5^; LNCaP and LNCaP (c+): 4×10^4^; and PC-3 and PC-3M: 5×10^3^), and grown for up to 2 weeks for colony formation. For the AR+ cells (VCaP, LNCaP, and LNCaP (c+)), the assay was performed in CSS, with or without 10 nM R1881. For the AR-cells (PC-3 and PC-3M), the assay was performed in fetal bovine serum. Colonies were fixed, stained, counted and imaged as previously described [30].

### Palmitate measurement

For the palmitate measurement, LNCaP and PC-3M cells were seeded in 24-well plates. LNCaP cells were treated with AdRSV or AdCav-1 for 24 hours and then with CSS, in the presence or absence of 10 nM R1881, for another 24 hours. PC-3M cells were treated with NCsi or Cav-1siRNA8 for 48 hours. Cells were washed with PBS, gently scraped, and then the pellet and supernatant were collected. Each cell pellet was added in 200 μL of methanol with palmitic acid-13C1 (0.5 ng; Sigma Aldrich, St. Louis, MO). The mixture was acidified with 1.0 μL of concentrated hydrochloric acid, vortexed with 3.0 mL of hexane, and centrifuged; the separated organic layer was evaporated. The dried extracts were subsequently derivatized using freshly prepared 1-(3-aminopropyl)-3-bromoquinolinium bromide [33], (Supplementary Materials and Methods).

### Transgenic PTENcKO mouse model

Female Pten^loxp/loxp^ mice with 129S4/SvJae BALB/c background from Jackson Laboratory were crossed with male PBCre4^+^ mice with C57BL/6 background from the NCI mouse repository to generate PBCre^+^;Pten^loxp/WT^ males and females, which were crossed to generate PBCre^+^;Pten^loxp/loxp^. Males from the last genotype were crossed with PBCav-1+ females of C57BL/6 background, which have been previously described by our team [21], to generate PBCre^+^;Pten^loxp/WT^;PBCav-1^−^, PBCre^+^;Pten^loxp/loxp^;PBCav-1^−^, and PBCre^+^;Pten^loxp/loxp^;PBCav-1^+^ mice (Supplementary Figure S2). The description of the genotyping and the transgenic animals used are presented in the Supplementary Materials and Methods.

### Immunohistochemistry

For the immunohistochemical analysis of Cav-1/ACC1/FASN pathway we used tissue sections from 12 benign normal prostate epithelia, 15 untreated and 15 ADT treated primary PCa and 8 PCa bone metastases. All the samples were from patients enrolled in a clinical trial conducted in MD Anderson Cancer Center. Specimens were processed as previously described [10], and antibodies against Cav-1 (sc-894, Santa Cruz), ACC1 (#3662, Cell Signaling), and FASN (Ab22759, ABCAM) were used to stain for Cav-1, ACC1, and FASN, respectively. For the evaluation of cancer incidence and AR, PCNA, Cav-1, ACC1, and cleaved caspase-3 staining in mouseVPs, mouse tissues were processed as previously described [21]; the following antibodies were used: Cav-1 (sc-894) and AR (sc-816) from Santa Cruz, ACC1 (#3662) and cleaved caspase 3 (#9661) from Cell Signaling, and PCNA (AV03018) from Sigma Aldrich.

### Measurement of long-chain fatty acids in BMA biopsy samples

For the measurement of long-chain fatty acids in human tissue samples, we included BMA from patients enrolled in the 2007-0590 clinical trial conducted at MD Anderson. Patients had established metastatic PCa progressed on systemic hormone therapy. We evaluated the BMA biopsy samples (5 ml) of 6 responders and 4 non-responders to AA at baseline and at 8 weeks of treatment. The designation of good versus poor clinical response was based on treatment status at 7.6 months, which was the median time on treatment among patients enrolled in the study. Therapy was discontinued at the treating physician's discretion in patients exhibiting disease progression. Samples were stored at −80°C. At the time of analysis, samples were extracted and prepared using Metabolon's standard solvent extraction method. The extracted samples were split into equal parts for analysis on the GC/MS and LC/MS/MS platforms, as previously described [34].

### Statistical methods

Data were summarized using descriptive statistics and exploratory graphics such as bar plots. For the in vitro experimental data unpaired t-tests were performed to compare between the treatment groups. In the experiments in which probability was determined, P values less than 0.05 were considered statistically significant, and all tests were 2-tailed. Synergism was determined using a 2-way analysis of variance (ANOVA) test as previously described [30]. For the immunohistochemical results, Wilcoxon rank-sum tests were used to compare the expression of different proteins between subgroups of tissue specimens. Spearman correlation was used for the correlation between Cav-1 and the ACC1 or FASN staining in mouse VPs. A Fisher analysis was used for the comparison of cancer or metastasis incidence in the transgenic mice.

## SUPPLEMENTARY MATERIALS AND METHODS


