# Supplementary material for: Caveolin-1 regulates hormone resistance through lipid synthesis, creating novel therapeutic opportunities for castration-resistant prostate cancer
Source: Oncotarget. 2016 Jun 16;7(29):46321–34. doi: 10.18632/oncotarget.10113 (PMC5216801; doi:10.18632/oncotarget.10113)
Supplement: Supplementary file 1 [file oncotarget-07-46321-s001.pdf]

# Caveolin-1 regulates hormone resistance through lipid synthesis, creating novel therapeutic opportunities for castration-resistant prostate cancer

## SUPPLEMENTARY MATERIALS AND METHODS

### RNA interference

Cav-1siRNA6 (SI00299614), Cav-1siRNA7 (SI00299621), Cav-1siRNA8 (SI00299628), FASNsiRNA1 (SI00059752), FASNsiRNA8 (SI00059759), ACACAsiRNA1 (SI00013622), ACACAsiRNA2 (SI00013629), and NCsiRNA (1022076) were purchased from Qiagen; ARsiRNA (s1538) was purchased from Life Technologies. Knockdown of Cav-1, FASN, ACC1, and AR was achieved by transiently transfecting the cells with a pool of Cav-1, FANS, ACC1, and AR-specific siRNA and a pool of non-targeting siRNA (NCsi) as the control, using lipofectamine RNAiMax transfection reagent (13778030, Life Technologies). PCa cells were seeded at the desired density (VCaP: 1.0 × 106; LNCaP and LNCaP (c+): 5×105; PC-3 and PC-3M: 2 × 105 in 6-well plates or 1/5 or 1/30 of AQ5 densities in 24- or 96-well plates, respectively). Cells were transfected with 20 nM Cav-1si, ACC1si, FASNsi, ARsi, or NCsi the following day. Twenty-four hours later, VCaP, LNCaP, and LNCaP (c+) cells were treated with CSS with 10 nM of R1881 or ethanol for another 24 hours. Protein extracts were prepared 48 hours after transfection.

### Palmitate measurement

For the palmitate measurement, LNCaP and PC-3M cells were seeded in 24-well plates. LNCaP cells were treated with AdRSV or AdCav-1 for 24 hours and then with CSS, in the presence or absence of 10 nM R1881, for another 24 hours. PC-3M cells were treated with NCsi or Cav-1siRNA8 for 48 hours. Cells were washed with PBS, gently scraped, and transferred to a 15-ml canonical tube. They were then spun at 12,000 rpm at ambient temperature for 5 minutes, and the pellet and supernatant were collected. Each cell pellet was added in 200 µL of methanol with palmitic acid-13C1 (0.5 ng; Sigma Aldrich, St. Louis, MO). The mixture was acidified with 1.0 µL of concentrated hydrochloric acid, vortexed with 3.0 mL of hexane, and centrifuged; the separated organic layer was evaporated. The dried extracts were subsequently derivatized using freshly prepared 1-(3-aminopropyl)-3-bromoquinolinium bromide [34]. An Agilent 6490 triple quadrupole mass spectrometer (Agilent, Santa Clara, CA) equipped with a Jet Stream electrospray ion source (ESI, Agilent), a 1290 Infinity ultra-high performance liquid chromatography system (Agilent), and MassHunter Workstation software (Agilent) was used to quantify palmitic acid. The chromatographic separation of palmitic, linoleic, and steric acids was conducted with a Chromolith C18 reverse phase column (50-2 mm) with a matching Chromolith guard column (5-2 mm) using 0.1% formic acid in water or 0.1% formic acid in methanol:water mobile phase gradient from 30% to 95% for 10 minutes. Fatty acids were introduced into the ESI source and analyzed in the positive ion mode. Molecular ion transitions (m/z 503.2 to 296.2 and 505.2 to 296.2) were monitored to quantify palmitate. Matching media (500 µL) were mixed with 500 µL of methanol containing internal standard before being prepared and analyzed in the same manner as the cell pellets.

### Transgenic PTENcKO mouse model

Female Ptenloxp/loxp mice with 129S4/SvJae BALB/c background from Jackson Laboratory were crossed with male PBCre$^{+}$ mice with C57BL/6 background from the NCI mouse repository to generate PBCre$^{+}$;Pten$^{loxp/WT}$ males and females, which were crossed to generate PBCre$^{+}$;Pten$^{loxp/loxp}$. Males from the last genotype were crossed with PBCav-1$^{+}$ females with C57BL/6, which have been previously described by our team [24], to generate PBCre$^{+}$;Pten$^{loxp/WT}$;PBCav-1$^{-}$, PBCre$^{+}$;Pten$^{loxp/loxp}$;PBCav-1$^{-}$, and PBCre$^{+}$;Pten$^{loxp/loxp}$;PBCav-1$^{+}$ mice (Supplementary Figure S1). For genotyping, tail DNA was subjected to a polymerase chain reaction analysis with the following primers: forward (5′-TCCCAGAGTTCATACCAGGA-3′) and reverse (5′-AATCTGTGCATGAAGGGAAC-3′); these were used to distinguish the wild-type and target alleles by amplifying the flanking loxP sites. The forward primer (5′-TTGCCTGCATTACCGGTCGATGCA-3′) and the reverse primer (5′-GATCCTGGCAATTTCGGCTAT-3′) were used to detect the PBCre transgene. The forward (5′-CCATGTTCATGCCTTCTTCT-3′) and reverse (5′-ATCGTAGACAACAAGCGGTA-3′) primers were used to detect the PBCav-1 transgene.

We used 128 transgenic animals in total for our studies. Particularly, we used 38 mice for the evaluation of PTEN and Cav-1 expression, 69 mice for the evaluation of cancer incidence, wet weight, and PCNA staining, and 21 mice for the evaluation of acute effects of castration. Mice were sacrificed using carbon monoxide euthanasia and decapitation.

## PBCre$^{+}$;Pten$^{loxp/loxp}$;PBCav-1 mouse model

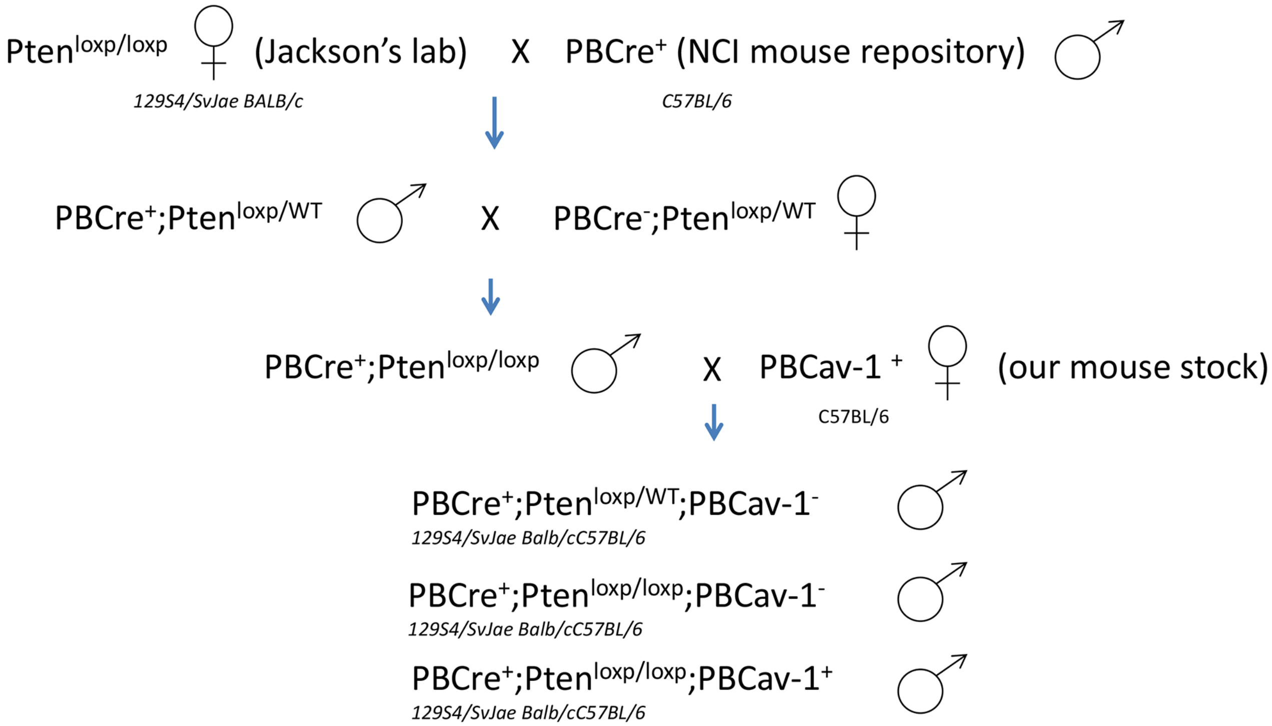


**Supplementary Figure S1: Development of the PBCre$^{+}$;Pten$^{loxp/loxp}$;PBCav-1$^{+}$ model.** Female Pten$^{loxp/loxp}$ mice from Jackson Laboratory with 129S4/SvJae BALB/c background were crossed with male PBCre$^{+}$ (NCI mouse repository) with C57BL/6 background to generate PBCre$^{+}$Pten$^{loxp/WT}$ males and females, which were crossed to generate PBCre$^{+}$Pten$^{loxp/loxp}$. Males from the last genotype were crossed with PBCav-1$^{+}$ females with C57BL/6 to generate PBCre$^{+}$;Pten$^{loxp/WT}$;PBCav-1$^{-}$, PBCre$^{+}$;Pten$^{loxp/loxp}$;PBCav-1$^{-}$, and PBCre$^{+}$;Pten$^{loxp/loxp}$;PBCav-1$^{+}$ mice.

| | | | | | | | |
|---|---|---|---|---|---|---|---|
| | **LNCaP cells** | | | | | | |
| | **Synergy** | **Control vs no R** | **Control vs AdCav-1** | **Control vs no R + AdCav-1** | **no R vs no R + AdCav-1** | **AdCav-1 vs no R + AdCav-1** | |
| **pAR ser81** | 0.0012 | <0.001 | 0.02 | 0.8 | <0.001 | 0.01 | |
| **AR** | 0.09 | <0.001 | 0.021 | 0.1 | 0.008 | 0.012 | |
| **ACC1** | 0.24 | 0.8 | 0.001 | 0.001 | <0.001 | <0.001 | |
| **FASN** | 0.24 | 0.001 | 0.023 | 0.006 | <0.001 | 0.016 | |
| | | | | | | | |
| | **VCaP** | | | | | | |
| | **Synergy** | **Control vs no R** | **Control vs Cavsi** | **Control vs no R + Cavsi** | **no R vs no R + Cav-1si** | **Cav-1si vs no R + Cav-1si** | |
| **pAR ser81** | <0.001 | 0.002 | 0.003 | <0.001 | <0.001 | 0.006 | |
| **AR** | <0.001 | <0.001 | 0.01 | <0.001 | <0.001 | 0.003 | |
| **ACC1** | 0.049 | 0.03 | <0.001 | <0.001 | <0.001 | 0.11 | |
| **FASN** | 0.14 | <0.001 | 0.003 | <0.001 | 0.002 | <0.001 | |
| | | | | | | | |
| | **LNCaP (c+)** | | | | | | |
| | **Synergy** | **Control vs no R** | **Control vs Cavsi** | **Control vs no R + Cavsi** | **no R vs no R + Cav-1si** | **Cav-1si vs no R + Cav-1si** | |
| **pAR ser81** | 0.0015 | <0.001 | 0.002 | <0.001 | 0.8 | 0.013 | |
| **AR** | 1 | 0.003 | <0.001 | <0.001 | <0.001 | <0.001 | |
| **ACC1** | 0.0085 | 0.008 | <0.001 | <0.001 | <0.001 | 0.3 | |
| **FASN** | 0.14 | 0.032 | 0.002 | <0.001 | <0.001 | 0.0013 | |
| | | | | | | | |
| | **LNCaP** | | | | | | |
| | **Synergy** | **Control vs AdCav-1** | **Control vs ARsi** | **Control vs AdCav-1+ARsi** | **AdCav-1 vs AdCav-1+ARsi** | **ARsi vs AdCav-1+ARsi** | |
| **AR** | 1 | 0.5 | <0.001 | <0.001 | <0.001 | <0.001 | |
| **ACC1** | 0.19 | <0.001 | <0.001 | 0.003 | 0.006 | <0.001 | |
| **FASN** | 0.08 | 0.025 | <0.001 | 0.006 | <0.001 | <0.001 | |
| | | | | | | | |
| | **PC-3** | | | | | | |
| | **NCsi vs Cav-1si6** | **NCsi vs Cav-1si7** | **NCsi vs Cav-1si8** | | | | |
| **ACC1** | <0.001 | 0.83 | <0.001 | | | | |
| **FASN** | <0.001 | <0.001 | <0.001 | | | | |
| | | | | | | | |
| | **PC-3M** | | | | | | |
| | **NCsi vs Cav-1si6** | **NCsi vs Cav-1si8** | | | | | |
| **ACC1** | <0.001 | <0.001 | | | | | |
| **FASN** | 0.002 | <0.001 | | | | | |

**Supplementary Figure S2: Determination of the synergy and statistical significance of the differences in protein expression of pARser81, AR, ACC1, and FASN in LNCaP, VCaP, LNCaP (c+), PC-3, and PC-3M cells on the basis of a Western blot analysis.** No R: androgen depletion.
